# Successful Treatment of Cardiogenic Shock Due to Takotsubo Cardiomyopathy With Left Ventricular Outflow Tract Obstruction and Acute Mitral Regurgitation by Impella CP

**DOI:** 10.7759/cureus.23168

**Published:** 2022-03-15

**Authors:** Ales Benak, Marek Sramko, Bronislav Janek, Michael Zelizko, Josef Kautzner

**Affiliations:** 1 Department of Cardiology, Institute for Clinical and Experimental Medicine, Prague, CZE

**Keywords:** left ventricular outflow tract obstruction, mechanical circulatory support, impella, cardiogenic shock, apical ballooning syndrome, takotsubo

## Abstract

Treatment of Takotsubo cardiomyopathy (TC) with left ventricular outflow obstruction (LVOTO) remains challenging. Mechanical circulatory support (MCS) as a bridge to myocardial recovery is sometimes the only therapeutic option, even though the optimal type of MCS is still under debate. This report describes a case of TC complicated by cardiogenic shock due to LVOTO and severe mitral regurgitation that was successfully treated with the latest generation percutaneous pump Impella CP®.

## Introduction

Takotsubo cardiomyopathy (TC) is an acute condition characterized by reversible left ventricular (LV) dysfunction with paradoxical apical ballooning and relatively preserved contractility of basal segments. It is often provoked by emotional or physical stress that leads to catecholamine surge, vasoconstriction of the coronary microcirculation, and myocardial stunning [1]. Although the disease has usually an uncomplicated course, 10%-15% of patients develop cardiogenic shock (CS) that can result from LV failure, dynamic LV outflow tract obstruction (LVOTO), or acute mitral regurgitation (MR) [2]. Treatment of these patients is challenging since inotropes are inefficient or even detrimental in the presence of LVOTO, diuretic therapy can worsen LV filling and ensuing LVOTO, and on contrary, fluid resuscitation can worsen MR and ensuing pulmonary edema. In such cases, mechanical circulatory support (MCS) remains the only therapeutic option.

Currently, veno-arterial extracorporeal membrane oxygenation (ECMO) is the most widely used MCS type in TC patients with CS, mainly because of its general availability [3]. However, ECMO can paradoxically worsen the LVOTO and LV contractility due to impaired LV filling and increased afterload. Percutaneous LV assist device Impella® (Abiomed Inc, Danvers, MA, USA) overcomes these limitations and thus better suits the specific pathophysiology of CS in TC. The latest generation Impella CP® provides up to 3.5 L/min of cardiac output and enables safe explantation using vascular closure devices. In this report, we present a case of a TC patient with CS due to dynamic LVOTO and acute MR who was successfully treated using this device.

## Case presentation

A 69-year-old woman with no history of cardiovascular disease was admitted to our cardiac intensive care unit for acute chest pain and dyspnea. The onset of symptoms was preceded by a sudden stressful situation (she had been frightened by a mailman ringing on the doorbell during her nap). At the initial examination, she was pale and clammy, dyspneic at rest with a SaO_2_ of 75%, and her blood pressure without vasopressors was 70/40 mmHg. Auscultation revealed a grade 4/6 systolic murmur over the whole precordium and rales over both lung bases. Lung congestion was also confirmed on X-ray. ECG showed sinus tachycardia of 110/min and 2-mm ST-segment elevations in V1-V3. She had increased high-sensitivity troponin T of 1,007 ng/L (normal value < 14 ng/L) and lactate of 32.4 mg/dL (normal value < 7.21 mg/dL), although B-type natriuretic protein, hepatic function tests, and renal function tests were initially in normal range.

**Figure 1:**
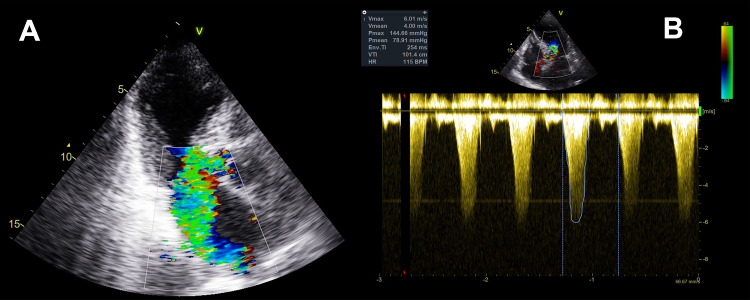
(a) Severe mitral valve regurgitation caused by the systolic anterior motion of the anterior leaflet of the mitral valve. (b) Severe left ventricular outflow tract obstruction (peak pressure gradient of 144 mmHg).

Bedside transthoracic echocardiography revealed the typical image of TC, with akinesis and ballooning of the apical half of the LV and ejection fraction (LVEF) of 30%-35%. Moreover, there was a 4/4 MR (Figure 1A) caused by systolic anterior motion (SAM) of the anterior mitral valve leaflet cusp and dynamic LVOTO with a peak pressure gradient of 144 mmHg (Figure 1B).

**Figure 2:**
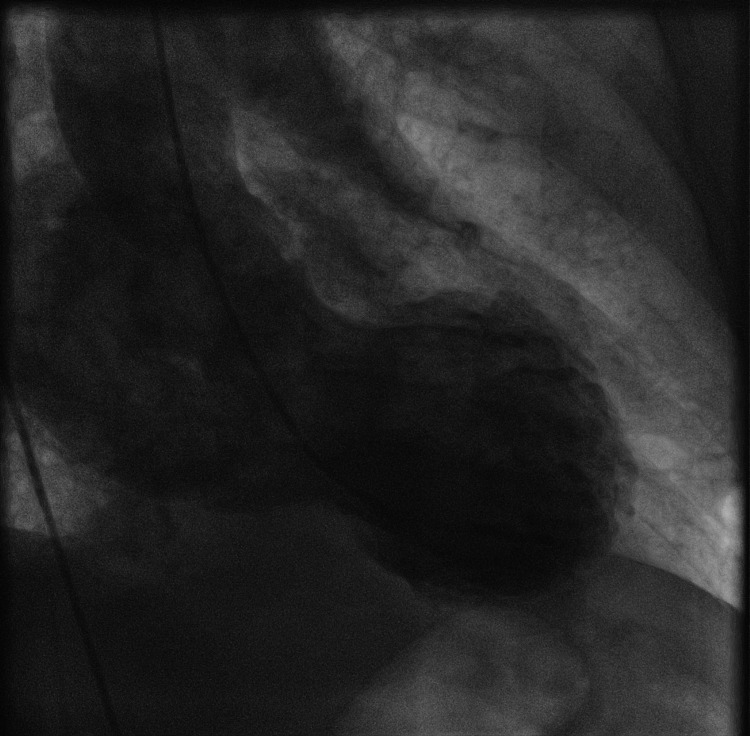
LV angiogram illustrating the typical finding of Takotsubo cardiomyopathy with severe mitral valve regurgitation.

Urgent coronary angiography excluded significant coronary artery stenoses, and left ventriculography confirmed the typical LV apical ballooning with grade 4/4 MR (Figure 2).

Because of persisting CS (SCAI C), we decided to implant an Impella CP® as a bridge to myocardial recovery. The device was inserted through the right femoral artery without complications, and the pump flow was set to P6 (2.3 L/min). At the time of active impella, the patient was anticoagulated with unfractionated heparin with a target activated partial thromboplastin time (aPTT) 65-85 s.

After transferring the patient to the cardiac intensive care unit, the patient condition rapidly improved, and pulmonary edema receded. Her blood pressure was stable without vasopressors, lactate levels normalized, and no inotropes or diuretics were needed. Echocardiography performed 48 hours later showed improvement of LVEF to 40% with absent SAM and MR was only mild. The pressure gradient in the LVOT could not be reliably measured. There was a drop in hemoglobin from 11 to 8 g/dL but without any recognizable source of bleeding or hemolysis. The levels of plasma-free hemoglobin remained normal (<6 mg/L), levels of lactate dehydrogenase were low (300-360 U/L) and the Impella® had an optimal position in the LV. Before the explantation, the patient tolerated the flow rate reduction to 1.5 L/min (P2) with no change in the arterial blood pressure. However, within a few hours after the removal of the Impella®, the patient developed mild pulmonary congestion and dyspnea, even though other hemodynamic parameters such as arterial blood pressure, oxygenation, and serum lactate remained stable. Echocardiography showed recurrence of LVOTO and a grade 2/4 MR due to SAM. Subsequently, we inserted a Swan-Ganz (SG) catheter to the pulmonary artery for hemodynamic monitoring and precise guidance of fluid therapy. After careful infusion of normal saline and two packed red blood cells, central venous pressure increased from 4 to 8 mmHg, pulmonary capillary wedge pressure decreased from 22 to 14 mmHg, and cardiac index increased from 2.4 to 2.6 L/min/min. The improvement in hemodynamics was accompanied by the disappearance of the LVOT and pulmonary congestion. Anemia did not recur. The patient was discharged on day 9 with a beta-blocker and angiotensin-converting enzyme inhibitor.

**Figure 3:**
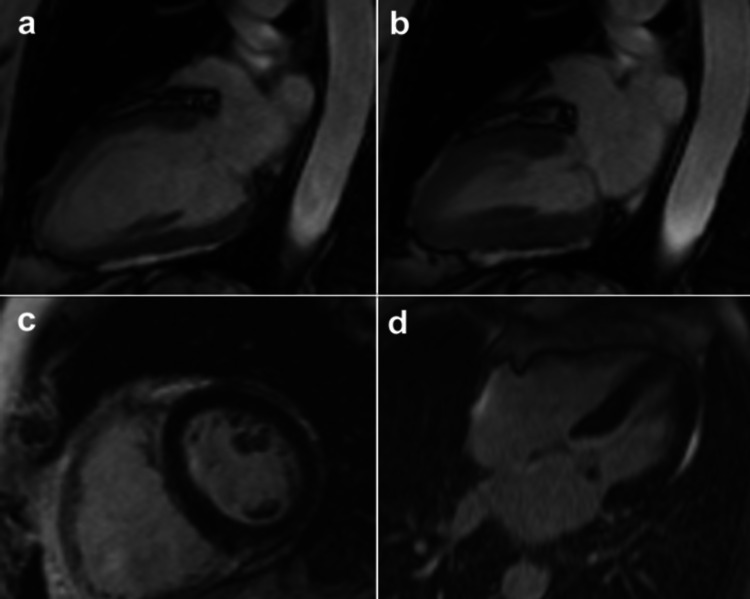
Cardiac magnetic resonance imaging proved normal function LV with no abnormalities in the LV kinetics (a, b) and no LGE (c, d). (a) Cine diastole, (b) cine systole, (c) LGE short axis, (d) LGE four chambers LGE - late gadolinium enhancement

Cardiac magnetic resonance imaging performed one month later found normal function and volume of the LV, with no abnormalities in the LV kinetics and no late gadolinium enhancement (LGE) (Figures 3a-3d).

Echocardiography showed a grade 1/4 MR and absent LVOTO. The patient remains asymptomatic six months after discharge.

## Discussion

CS associated with TC represents a serious complication and a major therapeutic challenge, especially when the CS is caused by LVOTO. Current treatment recommendations are based on clinical experience and expert opinions. As discussed earlier, the use of inotropes is not recommended in the presence of LVOTO [4]. Furthermore, volume expansion is problematic in the presence of pulmonary edema, diuretics or nitrates can exacerbate LVOTO, and beta-blockers are unsuitable for their hypotensive and negative inotropic effect. 

Short-term MCS can bridge the acute phase of CS and gain time until spontaneous myocardial recovery. In fact, the use of MCS in TC-related CS has been associated with lower in-hospital mortality [2]. Most centers use for this indication veno-arterial ECMO, mainly because it is the most widely available MCS for a relatively low cost. Nonetheless, the axial-flow LV pump Impella® may be a better choice of MCS type for CS with LVOTO, because, in contrast to ECMO, it does not impede the LV filling. This has been shown in two reports using successfully an earlier generation Impella 2.5® in patients with TC and LVOTO [5,6]. The latest generation Impella CP® provides even more powerful pump flow (up to 3.5 L/min) and includes a sheath that can reduce vascular access. The use of Impella CP® has been previously reported in four patients with TC and CS [3] although only one previously reported case had severe LVOTO and MR similar to our patient [7]. Our case report, therefore, contributes to the limited available experience with the use of Impella CP® in TC.

The optimal timing of MCS is still unclear. In our patient, we decided to explant the Impella® on day 3 because of unexplained continuous blood loss, which was in contrast to the stable hemodynamic condition. This was followed by transient recurrence of LVOTO, MR, and mild pulmonary congestion. Our experience suggests that in the absence of complications, the Impella® device should be left in place longer, even if the patient appears stable.

Lastly, adequate fluid management to optimize LV filling is crucial to the treatment of TC with CS [4]. Volume expansion can decrease LVOTO, but on the other hand, it can worsen pulmonary edema. Our case demonstrates the importance of precision fluid therapy guided by invasive hemodynamic monitoring with a Swan-Ganz catheter. Measurement of invasive hemodynamics can also be useful while weaning from MCS. In our case, we consider the delayed insertion of the Swan Ganz catheter to be a mistake.

## Conclusions

Impella CP® is a modern percutaneous MCS with the advantage of easy insertion and low risk of complications. Based on our experience, Impella CP® can be used effectively in the complex treatment of patients in CS in TC with LVOT and MR to bridge the acute phase and enable myocardial recovery.
